# ReActLab: A Custom Framework for Sensorimotor Experiments “in-the-wild”

**DOI:** 10.3389/fpsyg.2022.906643

**Published:** 2022-06-21

**Authors:** Priscilla Balestrucci, Dennis Wiebusch, Marc O. Ernst

**Affiliations:** Department of Applied Cognitive Psychology, Faculty for Computer Science, Engineering, and Psychology, Ulm University, Ulm, Germany

**Keywords:** remote experiments, engagement and user experience, actions and perception, sensorimotor adaptation, psychophysics

## Abstract

Over the last few years online platforms for running psychology experiments beyond simple questionnaires and surveys have become increasingly popular. This trend has especially increased after many laboratory facilities had to temporarily avoid in-person data collection following COVID-19-related lockdown regulations. Yet, while offering a valid alternative to in-person experiments in many cases, platforms for online experiments are still not a viable solution for a large part of human-based behavioral research. Two situations in particular pose challenges: First, when the research question requires design features or participant interaction which exceed the customization capability provided by the online platform; and second, when variation among hardware characteristics between participants results in an inadmissible confounding factor. To mitigate the effects of these limitations, we developed ReActLab (Remote Action Laboratory), a framework for programming remote, browser-based experiments using freely available and open-source JavaScript libraries. Since the experiment is run entirely within the browser, our framework allows for portability to any operating system and many devices. In our case, we tested our approach by running experiments using only a specific model of Android tablet. Using ReActLab with this standardized hardware allowed us to optimize our experimental design for our research questions, as well as collect data outside of laboratory facilities without introducing setup variation among participants. In this paper, we describe our framework and show examples of two different experiments carried out with it: one consisting of a visuomotor adaptation task, the other of a visual localization task. Through comparison with results obtained from similar tasks in in-person laboratory settings, we discuss the advantages and limitations for developing browser-based experiments using our framework.

## Introduction

In research fields such as experimental psychology and cognitive science, studies involving human participants are typically conducted in laboratory facilities with specialized equipment and under controlled conditions. In these settings, participants are usually recruited on-site among students or other interested visitors. Unsurprisingly, when the COVID-19 pandemic began in early 2020, the work pipeline revolving around this type of research was heavily disrupted, and researchers needed to adapt almost overnight to the unprecedented situation in order to continue collecting experimental data (Balestrucci et al., 2020).

While some researchers turned to online platforms for running psychology studies, the discussion around taking experiments outside of laboratories had started well before the pandemic. In fact, over the past two decades, different tools for hosting experiments and recruiting participants online have become more readily available to researchers (Reips, 2002; Behrend et al., 2011; Germine et al., 2012). Although experiments conducted online are less controlled than those in-person, studies have shown that migrating to such settings allows researchers to reach a larger, more diverse population, thereby leading to more generalizable and reproducible results, often in less time (Germine et al., 2012; Grootswagers, 2020). In many cases, hosting experiments on dedicated online platforms and recruiting participants *via* crowdsourcing services have represented a possible, and even advantageous, solution for conducting research (Berinsky et al., 2012; Crump et al., 2013; Semmelmann and Weigelt, 2017; Sauter et al., 2020). Moreover, the easy availability of portable devices, such as laptops, tablets, and smartphones, allows for greater flexibility in developing experimental frameworks outside of specialized facilities. Recently (Bedore et al., 2018) demonstrated that in certain cases using portable devices for visuo-motor experiments can produce results similar to those obtained using specialized equipment.

Several notable frameworks for conducting human-based research outside of specialized laboratories have been developed over the last few years, all of which present strengths and limitations. In particular, Takiyama and Shinya (2016) developed PoMLab (Portable Motor Learning Lab), a native application using the game development engine Unity, which can be downloaded on touchscreen devices and used for rehabilitation and clinical assessments. While the use of Unity and touchscreen devices allows for a robust approach, users must still download the application and calibrate it for a specific device, limiting the range of possible hardware platforms and therefore the number of users that can be reached. More recently, Tsay et al. (2021) developed OnPoint, a framework for web-based visuo-motor learning experiments deployed *via* crowdsourcing services, specifically Amazon’s Mechanical Turk and Prolific. In general, the use of web-based technologies guarantees a much higher degree of interoperability across platforms, however it presents limitations for certain visuomotor experiments. These issues are compounded with experiments embedded within the browser web page, where researchers are unable to control multiple components of the experiment. For instance, they cannot control, or even know, the type of hardware used by the participant. Likewise, participants must also necessarily make use of a mouse or trackpad to navigate the web page, which also leads to additional visual feedback for the experiment, namely the mouse cursor. Such artifacts likely produce ambiguous results. While these approaches focus mainly on bringing motor learning and adaptation studies outside of the lab, we instead seek a flexible platform with more finely grained control over multiple parameters, which would allow us to develop perceptual as well as motor experiments.

In this paper, we introduce ReActLab (Remote Action Laboratory), a framework for conducting experiments outside of laboratory facilities. ReActLab was originally intended for running studies on action and perception without relying on the highly specialized equipment usually available in experimental psychology laboratories; however, it can easily be customized to meet the design needs of researchers running a wide variety of experimental tasks (e.g., cognitive tasks or even online surveys). ReActLab is open-source, modular, and web-based, which makes it inherently cross-platform: it can be run on Windows, Linux, MacOS, and mobile devices, the only requirement is a modern and updated web browser.

Available for download to the research community, our framework can be used to design and run experiments by customizing template files included in the project repository, or by adding new functionalities to those already implemented. Experiments developed with ReActLab can be piloted and run by setting up a local server on the experimenter’s machine, or by setting up a server on dedicated machines or external cloud services. No software needs to be downloaded on the client machine, as experiments run exclusively in a web browser. In contrast to crowdsourcing alternatives, this setup allows for greater control over elements displayed to the user visiting the web page, such as implementing a full-screen viewport and eliminating the mouse cursor.

At the same time, by avoiding crowdsourcing alternatives we were necessarily limited in the number of participants we were able to recruit. Platforms such as Mechanical Turk allow for recruiting a larger and more representative population sample in relatively short time (Behrend et al., 2011; Johnson et al., 2021); however, such an approach necessarily leads to significant variability in the devices used by different participants. We instead decided to test our framework using only a specific model of Android tablet that we personally provided to participants. For this reason, we refer to our experiments as “remote” to distinguish them from fully online as well as typical in-person studies. To recruit participants, we advertised our experiments in the usual channels targeting university students and people from the local community as in typical laboratory experiments, and we arranged to meet participants briefly (and safely, given the regulations in place due to COVID-19) for the time necessary to exchange the equipment. We were thus able to achieve a compromise between the need to maintain social distance between experimenters and participants, while also still providing some form of standardization of the designed experimental procedure necessary for the proposed tasks.

While more information and technical details on how to use ReActLab can be found on the GitHub repository of the project, in our paper we provide a general overview of its current functionalities. We also present two experiments as a proof-of-concept for showing how ReActLab can be used in different scenarios: a visuomotor adaptation task and a visual localization task.

## The ReActLab Framework

ReActLab provides a template in JavaScript, HTML and CSS for designing behavioral experiments that can run in a web browser. It builds on the open-source runtime system Node.js, while rendering in the browser is performed using the phaser 3 framework. The HTML renderer pugjs is used to display forms and questionnaires that can be submitted to participants in various stages of the experimental session. The use of the pug renderer facilitates the implementation of questionnaires displayed in a browser since its templating mechanisms allow the reduction of code to a bare minimum, giving the developer the possibility to focus on actual questionnaire content. Data entered into the questionnaire is automatically saved by ReActLab, without requiring further programming.

**Figure 1:**
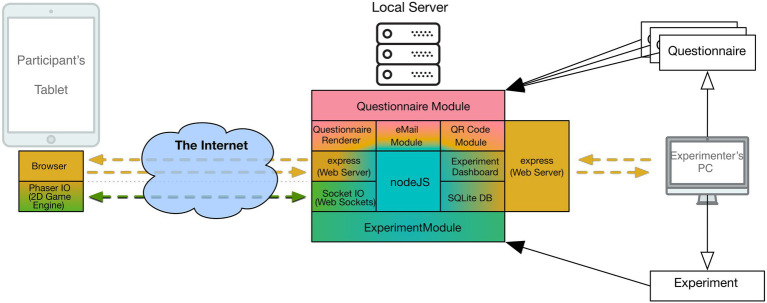
Schematics of the parts and modules in the ReActLab framework.

ReActLab makes use of other JavaScript modules in order to provide additional functionalities. At the time of writing, they consist in the following (Figure 1):

- E-mail module to manage invitations to the participants;
- QR Code generator to embed the participation link in the invitation email;
- SQLite database to store the collected data.

All functionalities can be accessed from the Experiment Dashboard, where experimenters can manage the tasks needed to run their experiment (e.g., generate new participation links and send invitation emails, retrieve and download collected data).

With ReActLab, all experiment code is located in one file on the server (or more, if the developer chooses to split code into multiple files). No additional code needs to be written for the client device, except if there are plans to extend the framework with additional remote procedure calls (see below). Using the existing functionality, code to be executed by the client is also specified within the experiment code on the server. Programming an experiment is thus largely reduced to implementing reactions to participant actions and storing data collected during trials.

Regarding storage, while the experiment is performed by a participant, their data is continuously updated and saved to the server. This prevents data loss in the case of an unexpected interruption of communication between server and client. Once any interrupted communication is resumed, the server communicates the most recently saved conditions to the client, allowing participants to continue the task from the last trial. All data is saved in lightweight formats such as plain text or comma-separated value (CSV) files, which makes the experiment database small and easy to work with on any platform. If researchers expect to collect large amounts of data, it is also possible to compress data before sending it to the server. As the capacity of our server has thus far exceeded our storage needs, we have not yet implemented such functionality, although it can easily be done.

Using the framework, it is also possible to allow multiple participants to simultaneously perform an experiment. To ensure that data from different participants is not overwritten, each participant is associated with a unique identification code.

In case participants wish to interrupt the experiment and withdraw their consent for the use of their experimental data, all files related to their participation are immediately deleted. In the current version of our framework, deletion must be performed manually by the experimenter. However, we intend to implement an automatic feature for deletion of withdrawn data in future releases.

### Designing Experiments With ReActLab

The only prerequisite for using ReActLab to design web experiments is an updated `node.js` release and the included package manager `npm`. These two components are also necessary to run the experiment, as all the source code for the experiment is stored on the server and there is no need to install specific programs or applications on the remote client (i.e., the browser of the device where the experiment runs). To download or view the code, see the project’s GitHub repository: https://github.com/CoPsLab/ReActLab.

This repository contains the framework project, a series of files and templates for creating custom web experiments in the JavaScript programming language, as well as the source code for the two experiments described in the next section. In this section, we give a brief overview of the main features that can be implemented with the provided templates. Please visit the ReActLab page on GitHub for additional information on installation and further technical details on how to use the framework.

#### Experiment

To create a new experiment in ReActLab, the easiest way is to duplicate the template-experiment.js file in the experiments folder, and rename it by substituting only the first part, <experiment-name>- experiment.js. This naming choice is important, as the -experiment.js file is used in the framework as the entry point for each experiment. While the main file is necessary to correctly run the experiment using the ReActLab framework, it is possible to distribute the code between different JavaScript files, if this facilitates writing and maintaining complex code.

The template file contains the structure needed to handle the main events of the experiments, such as establishing the connection between server and client, updating the internal counter that tracks the number of completed trials, showing visual stimuli and questionnaires, registering participants’ responses, and storing collected data in the database. Importantly, the experiment template automatically handles cases where the connection drops unexpectedly and allows for restarting the experiment from the beginning of the interrupted trial as soon as the connection is re-established, without introducing errors in the trial counter. Participants’ responses can be registered either with mouse or keyboard events. For mouse events, the coordinates in which the mouse is pressed (or released) are automatically expressed in a normalized space, in which height and width of the screen have length 1, and the origin is placed in the center of the screen. Touch events on touchscreens are also registered as mouse events and share the same properties. Visual objects that can be rendered during the experiment consist of preloaded images, called “assets” in the framework, and text. To avoid lag and delays caused by the communication between server and client, the temporalization of visual stimuli and other time-sensitive events can be handled locally on client as remote procedure calls. Simple functions, like visualizing or moving an object after a specified amount of time, are already available in ReActLab, while specialized operations can easily be added by extending the framework.

#### Questionnaires

HTML questionnaires and forms can serve various purposes throughout an experimental session. The example forms contained in the experiment folder of our repository show how to use questionnaires for some common cases:

- To register participants’ consent to data collection in the experiment. In our examples, the consent form questionnaire is submitted before starting the experiment. The consent to data collection is a mandatory field of the form and a requisite to start the experiment.
- To gather demographic information (e.g., sex, age, etc.). Within the same form, we included a field to compose a 6-digit anonymizing ID code. This code is automatically used to anonymize, store, and save all collected data, in order to separate experimental and demographic data from the code used to identify participants in the invitation email and to save consent data.
- To provide breaks between experimental blocks. In our examples, such intermediate forms did not include any questionnaire field, instead they encouraged participants to rest before the beginning of a new block of trials. However, such forms can be expanded by including fields to collect some responses (e.g., a qualitative rating relative to the previous experimental block).
- To collect feedback at the end of the experimental session. In our examples, most of the fields included in the final questionnaire are not mandatory. Nevertheless, the responses can provide a useful insight on how the task can be modified in order to make it more intuitive and easier to perform for participants.

In general, data collection *via* questionnaire is easy to implement using the modules included in ReActLab. However, it is also worth noting that the treatment of personal data is not directly enforced or even guided by the platform. In other words, experimenters themselves must follow the standards agreed upon and approved by the relevant ethical committee in order to avoid collecting sensitive or identifying data not explicitly stated on the consent form.

## Case Studies

In this section, we describe the implementation we used for running two different experiments with ReActLab outside of typical laboratory facilities. The first example consists in a visuomotor adaptation task, while the second is a perceptual visual localization task. Note that in this section, we emphasize demonstrating the methods used to collect meaningful data despite the limitations associated with the remote settings, rather than the novelty of the obtained results.

### Setup

Experiments were stored in our department server at Ulm University, and data was collected remotely on a 10.1-inch Samsung Galaxy tablet model A T510 (Samsung, Seoul, Korea). The tablet had a resolution of 1920 × 1200 pixels (screen dimensions: 217 × 135 mm). This tablet model was chosen because it has a relatively large screen and better video quality compared to other models in the same price range. We acquired several identical devices, allowing multiple authors to test and pilot the experiment before and during data collection. Due to lockdown regulations during the COVID-19 pandemic, authors were also able to use these devices while working from home, as well as hand them over to participants for the experiments themselves.

Since experiments were conducted in participants’ private homes, it was not possible to make experimental conditions homogeneous in terms of room lighting, sitting position, and distance from the screen. To mitigate the effects of these factors, we asked participants to follow instructions at the beginning of the experimental session about how to place the tablet and how to prepare the room for the experiment (e.g., by making sure that there would not be direct light hitting the tablet screen).

### Participants

We advertised experiments in the local community *via* university mailing lists, social media, and word of mouth. Interested participants were contacted by email, and an appointment was set up with the experimenter for handing off the equipment. Experimenters in charge of data collection disinfected the equipment before and after every exchange. The invitation email contained instructions on how to turn on the tablet and connect it to the local network, as well as a QR code. The QR code was scanned with the tablet’s camera to start the experiment. In some cases, the experimenter delivered the setup to the participant and did not remain with them for the duration of the experiment due to COVID-19-related distancing regulations. However, they were still reachable *via* telephone or email. Experimenters were also able to follow the completion of the task from a distance by monitoring the comment logs uploaded to the server, which occurred with minimal delay with respect to the events occurring in the browser. Every part of the experiment was designed to be as intuitive as possible for participants to carry out their tasks independently.

Participants were compensated for their participation (7.50 EUR/h). Experimental duration was kept below 90 min and limited to a single session. Before starting the experimental task, an informed consent form was submitted in accordance with the Helsinki declaration and the guidelines of the local ethics committee. After the consent form, participants completed a demographics questionnaire in which they also composed their own anonymizing ID code. All data collected from the experiment, except that related to the consent form, was saved using this ID.

During the session, prompts on the screen were displayed at regular intervals to encourage breaks, which were recommended but not mandatory. Instructions about the task were displayed several times during the session. The experiment terminated with a questionnaire in which participants were asked to provide feedback on their experience about taking part in the remote study. They could either fill in the digital form or give feedback during the debriefing session.

### Experiment 1: Adaptation to a Visuomotor Perturbation

Sensorimotor adaptation indicates our ability to gradually adjust and recalibrate the motor commands needed to perform a well-practiced action, in order to maintain adequate performance in response to changes in the environment or our own body (Shadmehr et al., 2010). Common examples of adaptation occur include adjusting to the visual distortions caused by new prescription lenses, learning to type on an unfamiliar keyboard, or experiencing changes in movement due to muscle fatigue.

Adaptation has been demonstrated in different motor domains, such as reaching (Krakauer and Mazzoni, 2011), walking (Torres-Oviedo et al., 2011), eye movements (Pélisson et al., 2010), speech production (Houde and Jordan, 1998), and in response to many different types of perturbation, such as visual rotations or displacements (Krakauer, 2009), force fields (Lackner and DiZio, 2005), and audio distortions (Keough et al., 2013). The first scientific studies on adaptation by German physiologist Von Helmholtz (1867) used prismatic lenses that displaced the visual field by several degrees, thus introducing a novel mapping between body movement (i.e., the arm reaching towards a target) and corresponding visual feedback. While prismatic lenses are still sometimes used to investigate the properties of adaptation, especially in clinical contexts (Morton and Bastian, 2004; Shiraishi et al., 2008), the majority of modern studies use computer tasks where a systematic perturbation is applied *via* software instructions. Adaptation has traditionally been theorized as a single process that can be described with a simple learning rule (Cheng and Sabes, 2006; Burge et al., 2008). More recent evidence, however, suggests that multiple, qualitatively distinct processes contribute to the error reduction measured in adaptation paradigms (Huberdeau et al., 2015; Krakauer et al., 2019). Along with an implicit component, likely dependent on cerebellar processes and outside of one’s awareness (Tzvi et al., 2021), recalibration to imposed perturbations also rely to some degree on overt cognitive strategies, mainly driven by the explicit knowledge of the presence of the imposed perturbation (Taylor and Ivry, 2012). Since implicit and explicit components both contribute to the measured adaptation response, distinguishing between their relative contribution is nontrivial. One way to mitigate the weight of cognitive strategies when studying sensorimotor adaptation is to introduce gradual, rather than large and abrupt, perturbations (Redding et al., 2005). To this end, Hudson and Landy (2012) demonstrated the feasibility of measuring adaptation by applying a sinusoidal offset. In their study, they showed that such a method provides advantages with respect to the classical introduction of a constant bias.

While perturbations introduced in experimental paradigms typically target a specific sensory channel (e.g., vision in prism adaptation, proprioception in force-field adaptation, etc.), it is important to remember that motor recalibration is driven by information from multiple sensory sources. This can lead to confounding factors introduced in sensorimotor adaptation experiments, as the response to the applied perturbation is masked or contaminated by other uncontrolled sensory information. This is the case, for example, when the participant’s hand is also visible during recalibration, as it also provides positional information in conflict with the feedback of the endpoint reaching movement (Henriques and Cressman, 2012; Barkley et al., 2014).

The aim of our experiment was to test whether an adaptation response to a visuomotor perturbation could be measured from data collected remotely. Given the setup, we recognized several methodological difficulties, such as the participant’s hand being close to the rendered stimuli, which could allow for comparison between the actual position of the pointing finger and the offset position of the perturbed feedback. While it is nearly impossible to completely avoid this issue, we reduced the contribution of this confounding factor by establishing as much distance as possible. Instead of allowing participants to perform their pointing movements on or around the exact coordinates of the target stimuli, they were only able to point within a limited lower frame of the touchscreen, while visual stimuli only appeared at corresponding coordinates in an upper frame. Moreover, to minimize explicit components of cognitive strategies, we adopted the method proposed by Hudson and Landy (2012) and introduced visuomotor perturbations that changed from one trial to the other only slightly following a sinusoidal function. In different blocks of trials, we tested different values of frequency of the sinusoidal function, ranging between one (slow perturbation) and four (fast perturbation) period repetitions per block. In accordance with Hudson and Landy (2012), we expected that the measured response would have the same frequency of the applied sinusoidal perturbation, but would differ from it in terms of amplitude and phase delay. In addition, we expected that amplitude and phase of the response would depend on the frequency of the perturbation: namely, we expected to find smaller amplitudes and greater delays as the velocity of the perturbation increased. To further characterize the results obtained remotely, we compared them with those from data obtained in an analogous adaptation experiment performed in our laboratory.

#### Participants and Procedure

Thirty-seven participants (age range: 18–58 years; 27 females, 10 males; 36/1 right/left-handed) volunteered in the remote adaptation experiment. One participant was excluded from further analysis as it appeared from the debriefing that she did not understand the instructions. All participants had normal or corrected-to-normal vision.

**Figure 2:**
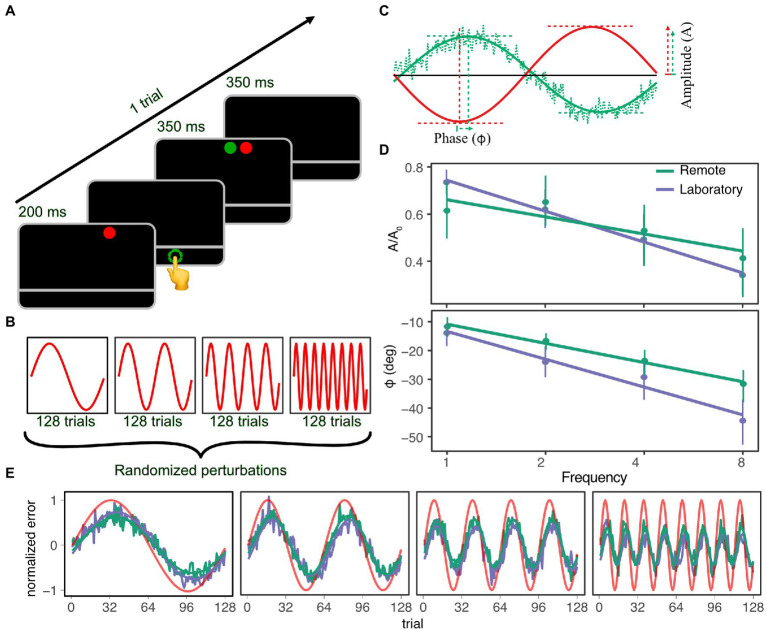
**(A)** Experimental procedure for each trial. **(B)** Perturbation in the four sequences of trials, each one characterized by a different frequency of the sinusoidal offset. **(C)** Definition of amplitude (A) and phase (*φ*) of the adaptation response (green line) with respect to the sinusoidal offset (red line). **(D)** Normalized amplitude (top panel) and phase (bottom panel) of the adaptation response as a function of frequency of perturbation for participants in the remote and laboratory setting (blue and purple symbols, respectively). Points represent estimates of the parameters as they are extracted from the model in Eq. 3.4, lines are bootstrap-based confidence intervals. **(E)** Average response of the two groups over trials in the four sequences. Perturbations are shown in red, and inverted for an easier reading of the representation.

The experiment was fully remote: participants completed the task independently in their own home, while the experimenter was available *via* telephone or email during the completion of the task. Participants were instructed to contact the experimenter at any time while completing the experiment, if they experienced any technical issues or required clarification regarding the task. The tablet was placed in landscape orientation on a desk in front of the sitting participant. At the beginning of each trial, a target (red circle, diameter 7 mm) appeared on the upper edge of the screen at a random horizontal coordinate and remained visible for 200 ms (Figure 2A). Participants were instructed to point on the edge of the screen closest to them at the horizontal location they perceived as corresponding to that of the target. A gray horizontal line on the black background was visible at all times, delimiting the area within which participants were allowed to point. Responses were considered as valid only after the target disappeared. Once pointing movement was registered, the target reappeared, together with the feedback stimulus (green circle, diameter 7 mm). Both target and feedback were visible for 350 ms, followed by an interval of 350 ms in which no stimuli were displayed and the tablet did not register any pointing. After this interval, a new trial started automatically.

On each trial (*t*), the feedback appeared at the same height of the target, while its horizontal coordinate (*F_t_*) corresponded to that of the hand pointing, *H_t_*, plus a perturbation $P_{t}:F_{t}=H_{t}+p_{t}$ .

Every 128 trials, the perturbation changed systematically according to a sine wave function:


(3.1) $$P_{t}=-A_{o}\sin\left[\frac{2\pi f}{T}\left(t-1\right)\right],$$

where *A_0_* = 22 mm was the amplitude of the sinusoidal perturbation, *f* was its frequency, defined as the number of times a complete cycle was repeated over the time unit *T = 128.* In accordance with the methodology proposed by Hudson and Landy (2012), the negative sign of the perturbation was introduced out of convenience for subsequent analysis, and the lag of one trial in the sine function was included so that the first trial of a sequence would have no perturbation. Participants performed the task in four sequences of trials, each one characterized by a different frequency of the sine perturbation: 1, 2, 4, or 8 cycles/sequence (Figure 2B). The order in which the sequences were presented was randomized across participants.

#### Data Analysis

To evaluate the adaptation response, we considered the motor error *X_t_*, defined as the difference between the horizontal coordinate of the pointing and that of the target: $X_{t}=H_{t}-T_{t}.$

The adaptive response to the sinusoidal perturbation in Eq. 3.1 is modeled with a sinusoidal function characterized by the same frequency of the perturbation, but with a smaller amplitude and a respective phase delay (Figure 2C):


(3.2) $$X_{t}=A\sin\left[\frac{2\pi f}{T}\left(t-1\right)+\varphi \right]$$

where *0 < A < A_0_* and *0 < ɸ < 2π.*

For the properties of trigonometric functions, the second term of Eq. 3.2 can be rewritten as follows:


(3.3) $$A\sin\left(\gamma_{t}+\varphi \right)=A\cos\left(\varphi \right)\sin\left(\gamma \right)+A\sin\left(\varphi \right)\cos\left(\gamma_{t}\right)$$

$$\mathrm{where}\;\;\;\;\;\;\gamma_{t}=\frac{2\pi f}{T}\left(t-1\right).$$

Because of the identity in Eq. 3.3, we can fit a linear mixed model (LMM) to the adaptive response


(3.4) $$X_{t}=\beta_{0}+\beta_{1}\sin\left(\gamma_{t}\right)+\beta_{2}\cos\left(\gamma_{t}\right)+Zu$$

Where *β_0_, β_1_, β_2_* are the fixed-effect parameters of the model, and *Zu* is the term accounting for the random effects. Parameter *β_0_* represents a constant response bias that changes randomly for every participant. Parameters *β_1_ and β_2_* are set to:


(3.5) $$\begin{array}{l} \beta_{1}=A\cos\left(\varphi \right),\\ \beta_{2}=A\sin\left(\varphi \right) \end{array}$$

By rearranging the terms in Eq. 3.5 we obtain amplitude and phase of the adaptation response. For the amplitude:


(3.6) $$\beta_{1}^{2}+\beta_{2}^{2}=A^{2}\left[\cos^{2}\left(\varphi \right)+\sin^{2}\left(\varphi \right)\right]$$

Given that $\cos^{2}\left(\varphi \right)+\sin^{2}\left(\varphi \right)=1$ , we obtain the amplitude of the fitted sinusoidal curve:


(3.7) $$A=\sqrt{\beta_{1}^{2}+\beta_{2}^{2}}$$

For the phase shift, given that $\tan\left(\varphi \right)=\frac{\sin\left(\varphi \right)}{\cos\left(\varphi \right)}$ , we obtain:


(3.8) $$\varphi =\tan^{-1}\left(\frac{\beta_{2}}{\beta_{1}}\right)$$

Eqs. 3.7 and 3.8 define the fixed-effect estimates of amplitude and phase. The parameter values at the individual participant level were calculated from the same equations, using the combination of fixed and random effects of the model.

We expected the values of both amplitude and phase to decrease nonlinearly as a function of the perturbation frequency. In addition to evaluating the trends for amplitude and phase in the remote setting, we wanted to compare such trends against those obtained in a more traditional laboratory environment. To do so, we considered a subset of data obtained in our laboratory at Ulm University from an analogous experiment on adaptation to sinusoidal offset. A total of ten participants (5 females, 5 males, age: 22.1 ± 2 years) were involved in the laboratory experiment, none of whom performed the task in the remote setting. While the methods used in the laboratory experiment were analogous to those described above, the setup differed: participants sat in front of a large LCD display (Sony 65X8505B, dimensions 144 × 81 cm, 3.840 × 2.160 pixels), kept their head on a chin rest, and performed their pointing movements with a stylus on a graphics tablet (WACOM Intuos 3 A3; active area 48.8 cm x 30.5 cm) placed on a desk between them and the display. The room’s lighting was turned off and participants were prevented from seeing their own hand by a black cloth fixed above the tablet. The number of trials as well as the frequency of the sinusoidal perturbations were the same in both experiments, but the amplitude of the perturbation differed because in the lab we were able to use a larger screen. The amplitude was set to *A*_0_ = 3 deg. In order to make the amplitude of the response comparable between the two experiments for the analysis, we normalized the response amplitude by the amplitude of the respective perturbation (*A*_norm_ = *A*/*A*_0_.)

In order to test whether the normalized amplitude and phase decreased similarly in the remote and laboratory setting, we fit the following LMM to both parameters:


(3.9) $$y=\delta_{0}+\delta_{1}\log\left(f\right)+\delta_{3}S+\delta_{3}S\log\left(f\right)+Zu$$

where *δ_0_,…, δ_3_* are the fixed-effect parameters of the model, *f* is the frequency of the perturbation, and *S* is the dummy variable coding for the setting (*S = 0* for the remote, *S = 1* for the laboratory setting.) All analyses were performed in R version 4.0.2 (R Core Team, 2020).

#### Results

The adaptation response to different sinusoidal perturbations was well described by functions with reduced amplitude and delayed phase with respect to the perturbation, both in a remote (Figure 2E, green line) and a laboratory setting (Figure 2E, purple line.)

The LMM in Eq. 3.9 showed a negative slope for the amplitude parameter (Figure 2D, top panel) as a function of frequency for both the group in the remote (−0.06, 95% confidence interval (CI): [−0.08, −0.04]) and in the laboratory setting (−0.13, 95% CI: [−0.17, −0.09]). The difference in slope between the groups was statistically significant (*χ*_1_ = 7.41, *p* = 0.006). For the phase parameter, the slope associated with the frequency was negative (−6.09, 95% CI: [−9.81, −2.18]) and statistically significant (*χ*_1_ = 8.81, *p* = 0.03), but there was no difference between groups (Figure 2D, bottom panel).

#### Discussion

In this first experiment, our aim was to evaluate the feasibility of using a small touchscreen tablet to study visuomotor adaptation in remote settings. Participants were able to adapt to a series of sinusoidal perturbations characterized by different frequencies in both the remote and laboratory group. In all cases, the adaptation response was well described by a sinusoidal function having the same frequency of the perturbation, but an amplitude reduction and a phase lag with respect to it. Moreover, amplitude and phase parameters decreased nonlinearly as a function of the perturbation frequency in both groups, despite some differences between groups in the rate of decrease, which were statistically significant for the amplitude parameter. Such differences are indicators that, despite the similarities, the settings where the task is performed are not interchangeable and may elicit slightly different processes. This could be due to the fact that the setups had different form factors: Not only was the tablet in the remote experiment much smaller than the screen used in the lab, but the pointing responses were collected on the same device, while the screen was perpendicular to the graphics device in the laboratory experiment. Moreover, despite the expedients put in place to give less weight to the vision of the hand, its contribution was not controlled as well as in the laboratory experiment, where the hand was hidden under a custom scaffold.

In their study, Hudson and Landy (2012) demonstrated that the adaptation response to sinusoidal offsets was detectable already for small perturbation amplitudes, while larger offsets were needed to detect a response when a constant step-function perturbation was used. As remote setups have typically smaller sizes compared to screens used in laboratories, having the possibility to detect an adaptation response already with small perturbation amplitudes can be especially beneficial when studies are performed in such conditions.

Even though participants in the remote group completed the task without direct supervision of an experimenter, the task was generally perceived as intuitive, and only in one case did a participant misunderstand the provided instructions. Like for online experimental studies, it is necessary to take into account that participants in remote samples may have different demographic characteristics than the typical attendants of experiments in laboratories (Henrich et al., 2010). Therefore, when designing the experimental tasks, as well as the instructions, it is important to consider factors such as a lack of familiarity with touchscreen devices, possibly linked to the fact that the age range of the participant sample can be wider compared to that obtained when recruiting participants among university students.

We can conclude that the method proposed in our remote experiment can be used to obtain reliable data on visuomotor adaptation. As instructions were generally clear even without the continuous, in-person supervision of an experimenter, this method can also be extended in fully online settings. In this case, it would be recommended to limit data collection to participants who have access to a relatively large touchscreen device, in order to use a fixed mapping between motor and feedback.

### Experiment 2: Visual Localization Task

When studying perception and action behavior, researchers are often interested in controlling and manipulating the perceptual certainty associated with the experimental stimuli (Rohde et al., 2016). Such manipulation is necessary, for example, when investigating how different sensory cues are combined together depending on their relative reliability (Ernst and Banks, 2002; Alais and Burr, 2004), or in order to assess changes in motor learning based on the uncertainty associated with the feedback about pointing performance (Burge et al., 2008; Van Beers, 2012).

A common way to change the perceptual certainty associated with a visual stimulus consists in blurring it by applying a Gaussian function. Using psychophysical procedures we can evaluate the relationship between the amount of blur of the stimulus, which is modulated by changing the standard deviation of the Gaussian function *σ_blur_*, and the ability to visually localize it, quantified by the just noticeable difference (JND) of the associated psychometric function. Typically, the JND increases monotonically, but nonlinearly, as a function of *σ_blur_*. In order to obtain a robust estimate of the relationship between perception and visual blur, it is recommended to control external sources of lighting in the environment, as differences in the visual contrast can easily modify perception. Hence, to obtain a significant increase in uncertainty, the rendered blur can become very large. Such potential limitations are easily overcome in laboratory settings, since they are designed to run experiments with minimal noise from environmental factors and using high-quality visual supports, such as large projection screens or large LCD displays. Rendering blurred visual stimuli on screen also offers the advantage that researchers can control the uncertainty associated with each stimulus independently. This is necessary when several levels of reliability need to be tested in rapid sequence, or when uncertainty is only coupled to certain stimuli and not others (Burge et al., 2008).

The motivation for our second experiment conducted outside of laboratory settings with ReActLab was to test viable methods for obtaining differences in the perceptual certainty associated with visual stimuli. Since the screen of the tablet that we used for the remote experiments was necessarily much smaller than typical devices used for the same purpose in the lab, we could not render stimuli with blurs large enough to induce a change in the JND. Therefore, we decided to physically blur the stimuli by providing participants with glasses containing an opaque layer blurring the entire visual field (and thus not only the stimulus but also the tablet itself.) In order to maintain flexibility in the design of experiments using our framework, we also wanted the ability to selectively blur only certain stimuli, while having the possibility to also show unblurred stimuli at times. To this end, we applied the blurring filter not to both eyes, but a blur filter on each lens separately, so that we could blur one eye while keeping the other unblurred. Additionally, the lenses of these goggles were anaglyphs, i.e., they consisted in a pair of complementary color filters (blue for one eye, red for the other), so that they selectively allowed viewing only specific light frequencies. By rendering stimuli on the tablet screen in the colors matching these filters, we could obtain objects visible to one eye while being transparent to the other. When applying a blurring layer to only one lens, we could effectively manipulate the uncertainty associated with different stimuli independently from each other.

#### Participants and Procedure

Fourteen participants (age range: 18–63 years; 7 females, 7 males; 13/1 right/left-handed) volunteered in this second remote experiment on localization. They all had normal or corrected-to-normal vision.

**Figure 3:**
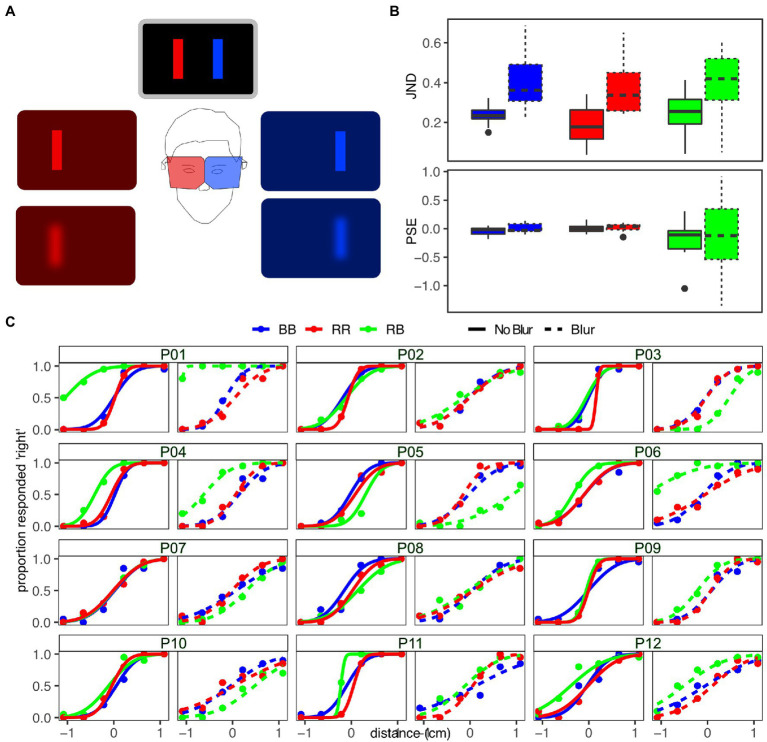
**(A)** Anaglyph glasses: filters for different colors (in our case, red for the right eye, and blue for the left eye) are applied to each lens, so that different stimuli are made visible for the two eyes, provided that the colors of the stimuli match those of the filters. The localizability of the stimuli can be modulated by applying a blurring layer on each filter. **(B)** Boxplots of the JND (top panel) and PSE (bottom panel) for the different conditions of color and blur. **(C)** Psychometric curves fitted for each individual participant in all conditions of color and blur.

Participants sat in a comfortable position in front of a desk, with the tablet lying in landscape orientation on a black cloth on a table. During the experiment, participants wore a pair of custom goggles in which the transparent lenses were replaced with two plastic optical filters: a red filter for the right eye, a blue filter for the left eye (Figure 3A). In cases where the COVID 19 conditions and pandemic regulations allowed, the experimenter stayed with the participant during the task and gave assistance and instructions about how to wear the goggles in the different experimental conditions. Visual stimuli consisted in blue and red rectangles on the black screen, and their color was chosen in such a way that the crosstalk and the ghosting effects between the two filters would be minimized. This ensured that the blue stimulus was only visible to the eye covered by the blue filter and transparent to the other eye and vice versa for the red stimulus (Figure 3A).

Using this setup, participants performed a two-alternative forced choice (2AFC) localization experiment. In each trial, two stimuli (a reference and a test stimulus) were presented briefly and in rapid temporal succession (time of presentation for each stimulus: 500 ms; inter-stimulus-interval: 500 ms), to avoid changes in fixation between the presentation of two stimuli. The reference stimulus appeared on the tablet at a random horizontal location, the test stimulus appeared at a distance of 2, 6.5, or 11 mm to the left or right of the reference. During the experiment, we modulated two different variables: (1) the color of the stimuli, and (2) the blur associated with them. We presented three different color conditions: (a) both reference and test stimuli were red (RR), therefore they were seen monocularly by the right eye; (b) reference and stimuli were blue (BB), so that they were seen only by the left eye; (c) reference stimulus was red and test stimulus was blue (BR - the reference stimulus was always seen through the right eye, the test stimulus through the left eye). In all conditions, the order in which reference and test stimulus were presented was randomized. The three color conditions were repeated under two different blur conditions: one in which no blur was applied to the visual stimuli, the other in which stimuli were viewed through a blurring layer. The blurring effect was obtained by adding a layer on each of the filtered lenses.

In total, each participant completed 720 trials of the 2AFC task (20 repetitions x 6 distances x 3 colors x 2 blurs). Participants performed the task first without blur, followed by the blurred conditions. The order of presentation of the three blocks with the different color conditions was randomized in each sub-section.

#### Data Analysis

For each condition of color and blur, we fitted a psychometric curve to the binomial responses of individual participants and extracted two descriptive parameters for the curve (Moscatelli et al., 2012): just noticeable difference (JND) and point of subjective equivalence (PSE). We then performed 3 × 2 repeated measures ANOVAs (3 colors x 2 blurs) on both parameters. Greenhouse–Geisser correction was applied if data violated the sphericity assumption, and significance of effects was set at *p* = 0.05 for all analyses. The models were visually inspected to ensure normality of the residuals.

#### Results

For the JND (Figure 3B, top panel), we found a significant effect of blur (*F*_1,11_ = 35.21, *p* < 0.001), with the blurred condition associated with significantly larger values of JND. Neither the main effect of color nor the interaction between color and blur was significant (all *F* ≤ 1.38, all *p* ≥ 0.27).

For the PSE, we found no significant main effect or interaction (Figure 3B, bottom panel; all *F* ≤ 1.61, all *p* ≥ 0.23). For individual psychometric functions (Figure 3C), it should be noted that there is a large variability between individuals concerning the bias of the function in RB conditions, especially in the one with blur.

#### Discussion

Using our remote methodology, we could replicate a well-known effect in visual perception: namely, modulating the blur associated with visual stimuli affected the ability to precisely localize them (Rohde et al., 2016). This resulted in a statistically significant difference between the JNDs of the blurred and unblurred conditions. Importantly, the effect was unaffected by the color of the visual stimuli, both when reference and test stimuli had the same color (i.e., both reference and test stimulus were blue/red) as well as in the dual color condition. While this aspect would not be considered in typical setups, it becomes relevant with the methodology proposed, as each stimulus color was only visible through the lens of one eye, while being filtered out in the other eye. Since blurring the visual stimuli affected the precision with which they could be localized, we did not expect that a systematic bias would be introduced, which would translate in a change of the PSE associated with the different conditions. In fact, there were no significant main effects or interaction for the PSE associated with the experimental variables. However, from a closer inspection of the psychometric curves at the single-participant level (Figure 3C), we can see that the curves fitting the response in the dual-color conditions (green lines) are shifted to the left or right with respect to the other two in several cases, especially for the blurred condition. In other words, the PSE associated with the dual-color condition has a larger variability between participants compared to the same-color conditions. This effect is due to the fact participants see the stimuli of different colors through the anaglyph glasses, and since this task was focused solely on testing differences of JND, we avoided the introduction of references that could facilitate stimulus localization. This setup caused ambiguity in selecting a point of fixation, which explains the variability between participants, and the fact that this is especially evident in the blurred condition. It is worth taking into account this effect induced by the use of anaglyph lenses when designing future experiments in which the introduction of a systematic perceptual bias can affect the results of the phenomena under investigation. One possible way to mitigate the lack of a stable fixation point might consist, for example, in periodically showing a fixation cross.

Due to the use of the custom anaglyph glasses, this type of experiment can hardly be implemented in a fully online procedure, unless some organization is put in place to ship the equipment to participants. However, this approach could be very advantageous when conducting tests with hospitalized patients.

While further testing is still needed, the reported experiment served as a preliminary proof-of-concept for the proposed method. Given the obtained results, we can see possible directions for future experiments. First, we could improve the task by adding fixation control, and second, we could test participant perception with more combinations (e.g., blurring only one eye). Once the method is fully tested, it could be applied to more complex experiments such as in combination with a sensorimotor adaptation task.

## General Discussion

To demonstrate the functionalities of ReActLab, our newly developed framework for browser-based studies, we presented two different experiments: a visuomotor adaptation task and a visual localization task. In both cases, the data collected remotely were both in line with our expectations and qualitatively similar to those obtained in typical laboratory settings. These findings demonstrate that ReActLab is a valid and viable tool for running experiments remotely. Beyond achieving near parity in results, more generally the ReActLab framework also provides several technical advantages for designing and running experiments. First, since the modules that provide the different functionalities in ReActLab are written in JavaScript, the software necessary to run experiments is fully browser based. It is therefore easy to re-use and modify scripts in order to target different kinds of hardware, such as migrating an experiment from a desktop computer to a touchscreen device or vice versa. Second, the libraries included in ReActLab are all open source, currently maintained, and widely used in the web development community. Maintaining and updating the framework thus becomes a question of simply updating to the latest version of `node` and the `npm` package manager. Experiments can continue to run on any device, as long as the device has an updated browser installed, and a stable internet connection is available. Lastly, ReActLab is easily extensible by writing custom code or incorporating different available modules in the already existing framework. The framework is therefore easily adaptable to situations and research requirements which were not originally considered. For example, we currently plan to extend the functionalities of the framework by introducing two new modules: one for presenting auditory stimuli and another for multi-participant experimentation. Another planned improvement will consist of allowing the experimenter to follow the participant’s progress while not present in person, not only *via* console logs but also by mirroring the participant’s screen on an additional read-only device.

Due to its stage of development and the nature of web-based experimentation, several limitations should be noted. Since the project is still in its early stage of development, we have not yet developed a user-friendly interface for ReActLab. As a result, some knowledge of web-based programming is needed to use the framework to its fullest extent. While other web-based research frameworks such as Pavlovia (Peirce et al., 2019) or Jatos (Lange et al., 2015) provide a more user-friendly interface for setting up web experiments, ReActLab offers more flexibility at the expense of a steeper learning curve for the initial setup. Researchers planning web-based experiments should also keep in mind that, due to the nature of working with the browser, and in particular with JavaScript, it is difficult to fully control the timing of events occurring on the client machine while running the experiment (Anwyl-Irvine et al., 2020.) This can result in less temporal precision compared to what can be achieved with specialized laboratory setups. We have attempted to mitigate this effect by limiting client–server communication for time-sensitive tasks (e.g., the timing of stimulus presentation or the acquisition of participant’s reaction times.) Nevertheless, possible timing delays cannot be completely ruled out, and they should be taken into consideration when programming experiments requiring very fine temporal tuning.

Despite such limitations, we hope that such research around alternative approaches to conducting behavioral experiments will prove to be beneficial, not only for establishing continuity in our own work during the pandemic, but also for facilitating future experiments in different settings, such as in hospitals, clinics, or schools. Given its portability, maintainability, and extensibility, as well as the ability to approximate results obtained with specialized laboratory equipment, ReActLab provides one such feasible alternative for research on action and perception.

## Data Availability Statement

The raw data supporting the conclusions of this article will be made available by the authors, without undue reservation.

## Ethics Statement

The studies involving human participants were reviewed and approved by Ethikkommission der Universität Ulm. The patients/participants provided their written informed consent to participate in this study.

## Author Contributions

PB and MOE: formulated overarching project aims. DW: conceived the software framework. DW and PB: tested and developed the software. PB and MOE: collected and analyzed data. PB: wrote original draft. PB, DW, and MOE: reviewed and edited manuscript. All authors contributed to the article and approved the submitted version.

## Funding

Funded by the Deutsche Forschungsgemeinschaft (DFG, German Research Foundation) – Project-ID 251654672 – TRR 161.

## Conflict of Interest

The authors declare that the research was conducted in the absence of any commercial or financial relationships that could be construed as a potential conflict of interest.

## Publisher’s Note

All claims expressed in this article are solely those of the authors and do not necessarily represent those of their affiliated organizations, or those of the publisher, the editors and the reviewers. Any product that may be evaluated in this article, or claim that may be made by its manufacturer, is not guaranteed or endorsed by the publisher.
